# Effects of tailored telemonitoring on functional status and health-related quality of life in patients with heart failure

**DOI:** 10.1007/s12471-019-01323-x

**Published:** 2019-08-14

**Authors:** A. J. Gingele, B. Ramaekers, H. P. Brunner-La Rocca, G. De Weerd, J. Kragten, V. van Empel, K. van der Weg, H. J. M. Vrijhoef, A. Gorgels, G. Cleuren, J. J. J. Boyne, C. Knackstedt

**Affiliations:** 1 Department of Cardiology, Maastricht University Medical Centre, Maastricht, The Netherlands; 2 Department of Clinical Epidemiology and Medical Technology Assessment, Maastricht University Medical Centre, Maastricht, The Netherlands; 3 Department of Cardiology, Zuyderland Hospital, Sittard, The Netherlands; 4 Department of Cardiology, Zuyderland Hospital, Heerlen, The Netherlands; 5 Department of Patient and Care, Maastricht University Medical Centre, Maastricht, The Netherlands; 6 Panaxea b.v., Amsterdam, The Netherlands; 7 Department of Family Medicine and Chronic Care, Vrije Universiteit Brussel, Brussels, Belgium; 8 CAPHRI, Department of Health Services Research, Maastricht University, Maastricht, The Netherlands

**Keywords:** Telemedicine, Heart failure, Health-related quality of life

## Abstract

**Background:**

Functional status and health-related quality of life (HRQoL) are important in patients with heart failure (HF). Little is known about the effect of telemonitoring on functional status and HRQoL in that population.

**Methods and results:**

A total of 382 patients with HF (New York Heart Association class 2–4) were included in a randomised controlled trial to investigate the effect of tailored telemonitoring on improving HRQoL and functional status in HF patients. Randomisation was computer-generated with stratification per centre. At baseline and after 12 months, patients’ functional status was determined by metabolic equivalent scores (METS). HRQoL was measured with the EuroQol five dimensions questionnaire (EQ-5D), visual analogue scale (VAS) and Borg rating of perceived exertion scale (Borg). Additional outcome data included number of HF-related outpatient clinic visits and mortality. Telemonitoring was statistically significantly related to an increase in METS after 1 year (regression coefficient 0.318; *p* = 0.01). Telemonitoring did not improve Borg, EQ-5D or VAS scores after 1 year. EQ-5D [hazard ratio (HR) 0.20, 95% confidence interval (CI) 0.07–0.54], VAS (HR 0.98, 95% CI 0.96–0.99), Borg (HR 1.21, 95% CI 1.11–1.31) and METS (HR 0.73, 95% CI 0.58–0.93) at baseline were significantly associated with survival after 12 months.

**Conclusions:**

Tailored telemonitoring stabilised the functional status of HF patients but did not improve HRQoL. Therefore, telemonitoring may help to prevent deterioration of exercise capacity in patients with HF. However, because our study is a reanalysis of a randomised controlled trial (RCT), this is considered hypothesis-generating and should be confirmed by adequately powered RCTs.

**Electronic supplementary material:**

The online version of this article (10.1007/s12471-019-01323-x) contains supplementary material, which is available to authorized users.

## What’s new?

- Tailored telemonitoring increases knowledge and self-care in heart failure patients.
- Tailored telemonitoring seems to be a reasonable intervention to improve functional status in patients with heart failure.
- Functional status and quality of life at baseline are suitable predictors for 1‑year survival in heart failure patients.

## Background

Heart failure (HF) is a chronic disease with high prevalence [1]. It is a costly and disabling disorder with poor prognosis and high mortality [2]. Moreover, patients with HF experience fatigue on exertion and have a decreased health-related quality of life (HRQoL), the latter being associated with increased mortality and morbidity [3]. Good functional status is an important prognostic marker in patients with HF [4].

One of the means currently used to follow up patients is telemonitoring, which consists of several invasive and non-invasive techniques and can be used for structured follow-up of HF patients [5]. Telemonitoring may reduce mortality and improve QoL in patients with HF [6].

However, numerous different invasive and non-invasive telemonitoring systems are available to assess clinical variables of HF patients. Different non-invasive telemonitoring systems are comparable regarding measurement of blood pressure, pulse and body weight but differ in additional functions [5]. This impedes a comparison between systems and results might depend on the individual system.

The telemonitoring in heart failure (TEHAF) trial used a patient-driven tailored telemonitoring device, the Health Buddy [7]. Besides detecting patients’ clinical deterioration, the Health Buddy supports patients in improving their knowledge of HF. It has been shown that use of the Health Buddy increases disease-specific knowledge and self-care abilities in HF patients [7]. Other studies have pointed out that an increase in self-care abilities may be associated with higher functional status and QoL [8, 9]. Despite the evidence that telemonitoring may improve self-care, little is known about the influence of telemonitoring on functional status in HF patients.

Therefore, this reanalysis of the original TEHAF trial elaborates the hypothesis that tailored telemonitoring may positively influence functional status and HRQoL in patients with HF. Additionally, we investigated if functional status and HRQoL at baseline are suitable variables to predict survival in HF patients.

## Methods

### Study design

**Fig. 1:**
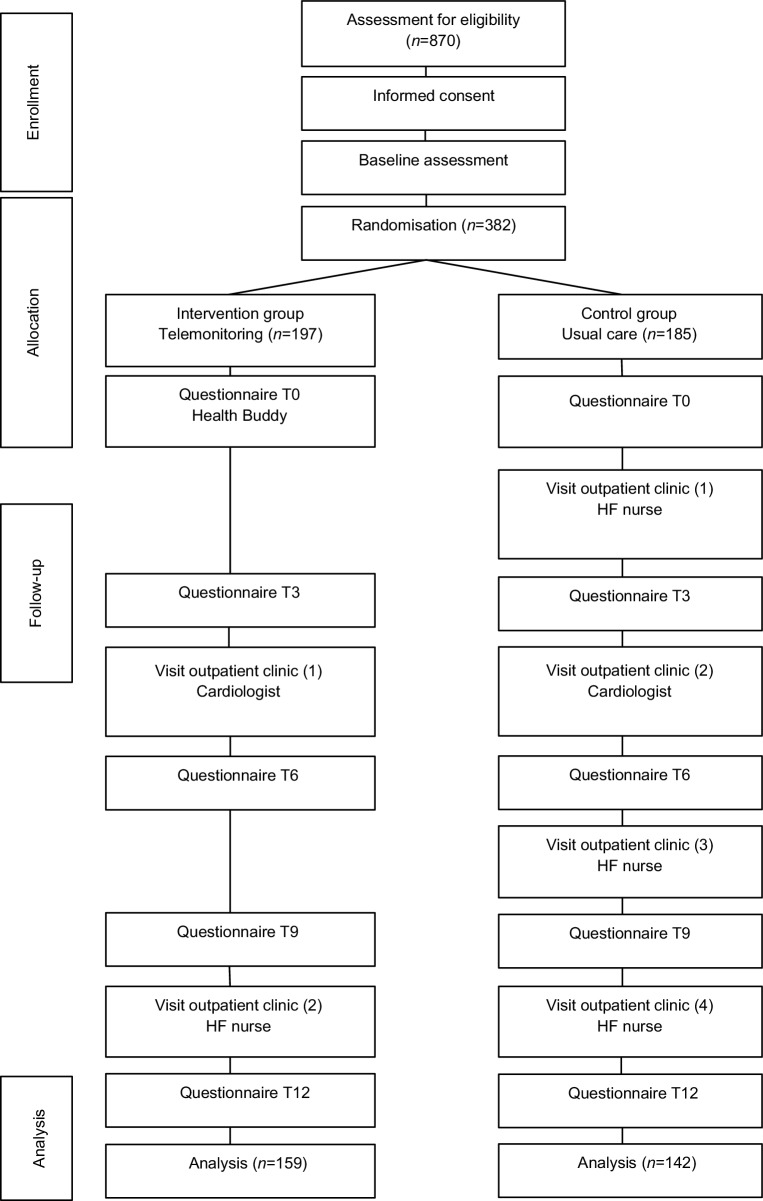
Study design of the telemonitoring in heart failure (TEHAF) trial. *HF* Heart failure

The TEHAF study, a randomised controlled trial performed between 2007 and 2010, compared tailored telemonitoring using the Health Buddy to usual care in HF patients [10]. Further study details have been published elsewhere [7]. In brief, HF patients with New York Heart Association (NYHA) functional class 2–4 that were treated by a cardiologist and were in the care of a heart failure nurse (HFN) were included. HF was defined as ‘at least one episode of fluid retention requiring diuretics, either with an echocardiographic left ventricular ejection fraction ≤40% or a preserved ejection fraction with diastolic dysfunction’ [7]. Patients were included in the study either during a visit to the outpatient clinic of one of the participating centres (Sittard, Heerlen, Maastricht) or when they were visited by a HFN, in the home situation. Exclusion criteria were: unable to give informed consent, visual impairment, hearing impairment in combination with living alone, no command of the Dutch language, planned hospital admission within 3 months and/or chronic obstructive pulmonary disease, Parkinson’s disease, extracorporeal dialysis, (pre)dementia or another disease with an expected shortened lifespan. A total of 382 patients were included. The trial was completed by 301 patients (79%). All patients who did not finish the study were excluded from all further analyses. The follow-up period was 12 months. Before randomisation, written informed consent was obtained. Randomisation was computer-generated with stratification per centre. Four times during the study, data on patients’ self-care behaviour (measured with the European Heart Failure Self-Care Behaviour Scale) [11], self-efficacy (measured with the Barnason Efficacy Expectation Scale) [12] and compliance (measured with the Heart Failure Compliance Scale) [13] were collected (Fig. 1).

### Objectives and endpoints

The primary objective of our reanalysis of the TEHAF study was to assess the effect of tailored telemonitoring on functional status, defined as change in metabolic equivalents (METS) scores, comparing an intervention group and a control group.

The secondary objective was to assess the effect of tailored telemonitoring on HRQoL in HF patients, defined as change in Borg rating of perceived exertion scale (Borg), visual analogue scale (VAS) and EuroQol five dimensions questionnaire (EQ-5D) scores during follow-up, comparing an intervention group and a control group. Additional secondary endpoints were mortality rate, number and length of HF-related hospital admissions, and number of outpatient clinic visits due to HF during 1 year of follow-up.

### Data collection

At baseline and after 12 months, patient characteristics, Borg, METS, VAS and EQ-5D scores were obtained as described below. Additionally, clinical data about mortality, number and length of hospital admissions and number of outpatient clinic visits due to HF were collected after 12 months.

### The Health Buddy

The Health Buddy, an electronic device consisting of a display screen and four buttons, was assigned to the intervention group. On a daily basis, patients received disease-specific education and self-care support, as well as questions about their symptoms, knowledge and behaviour. First, all collected data were sent to a protected server. Then, the HF specialist could see the data via an i‑Care desktop. Based on the answers given, patients were classified into low-, medium- or high-risk profiles for clinical deterioration. If necessary, patients were contacted for further analysis and support. Every 3 months, and in-between if necessary, questions were readjusted on an individual basis. For example, patients with frequent hospital admissions were allocated to a program that focused more on symptom monitoring, whereas patients with a low disease-specific education level were allocated to a program that contained a highly intensive education program. European Society of Cardiology guidelines were recommended to treat patients in both groups [10].

### Metabolic equivalents

METS describe the amount of energy used to perform a physical task at a certain level of intensity. It indicates patients’ global cardiopulmonary fitness. In this study, METS were obtained by using a dedicated questionnaire consisting of 39 items (see Electronic Supplementary Material). Patients were asked about their capability to and limitations in performing certain physical tasks. The more demanding the task, the higher the METS obtained [14]. Of all performable tasks, the one with the highest METS was regarded as the reference value for that person’s cardiopulmonary fitness.

### EQ-5D and VAS

EQ-5D is a scoring system to assess HRQoL. It consists of a questionnaire and a VAS. Using the questionnaire, patients’ current health status is depicted with regard to five different domains: mobility, self-care, usual activities, pain/discomfort and anxiety/depression. Based on the answers given, utility scores can be calculated to describe HRQoL. Utility scores can vary from −0.59 (‘health status worse than death’) to 1 (‘perfect health’) [15].

The VAS consists of a vertical line displaying values from 0 to 100, where 100 stands for ‘best imaginable health state’ and 0 represents ‘worst imaginable health state’ [16].

### Borg scale

Borg is a diagnostic test used to rate the level of exertion of an individual. It consists of a questionnaire that can be used to evaluate the effort when performing a certain task [17, 18]. In the present study, the key question asked was: ‘How exhausting for you is taking a shower or bath?’ Potential answers and thus scores ranged from 6 (‘no effort’) to 20 (‘maximal effort’).

This study was approved by the local ethics committees of Maastricht (METC 15-4-195.1/ivb) and Heerlen/Sittard [METC 15-N-188(15-4-195)] University. The investigation conforms to the principles outlined in the Declaration of Helsinki [19].

### Statistical analysis

Descriptive statistics were used to describe the baseline characteristics of the population, stratified by treatment group. To analyse differences in baseline variables, Student’s *t*-test (if data were normally distributed) or the Mann-Whitney test (if data were not normally distributed) was used for continuous variables and χ^2^ for discrete variables. Single stochastic regression imputation [R code: mice(data, method = ‘pmm’, *m* = 1, maxit = 1, seed = 1)] was used to impute missing values for (total number of missing values in brackets): ejection fraction (4), duration of HF (19), pacemaker (13), NYHA class (12), marital status (2), educational level (16), left bundle branch block (6) and cause of HF (3) [20]. Results shown are with imputed numbers only. Excluding patients in whom some values were missing did not change the results (data not shown).

Linear regression was used to examine the impact of the following baseline covariates on HRQoL (i.e. EQ-5D utility score, VAS score, BORG score and METS score) measured 1 year later: baseline HRQoL, treatment group, ejection fraction, duration of HF, NYHA functional classification, pacemaker, marital status, age, sex, educational level.

Interaction terms were tested and forward selection was used to select covariates/interaction terms [using the stepAIC() function in R] using a *p*-value of 0.05 as selection criteria [*k* = qchisq (0.05, 1, lower.tail = *F*)].

Additionally, regression techniques were used to examine the impact of baseline HRQoL (i.e. EQ-5D utility score, VAS score) and of functional parameters (BORG score and METS score) on overall survival, number and length of hospital admissions and number of outpatient clinic visits due to HF (all after 1 year). Cox regression was used to analyse overall survival.

A *p*-value of 0.05 or less was considered to be statistically significant. All analyses were performed in R version 3.1.3.

## Results

Baseline characteristics are shown in Tab. 1. An elderly HF population with mild to moderate symptoms was included. Regarding the questionnaire, 86% of all patients completed the EQ-5D and VAS, 88% the METS and 86% the Borg at both time-points. Follow-up was incomplete in 81 patients (21%), 38 in the intervention arm versus 43 in the usual-care arm. Reasons for incomplete follow-up were death (18 vs12), increasing physical impairment (13 vs 10), stress or losing motivation (5 vs 14), other (2 in both groups) or lost to follow-up (5 in the control group).

**Table 1:** Baseline characteristics of study population

Variable	*n*	Intervention (197)	Control (185)	*p-*value
*Age*	382	71.0 ± 11.9	71.9 ± 10.5	0.621
≥75		88 (44.7)	85 (46.5)	0.199
*Gender*	382			0.747
Male		115 (58.4)	111 (60.0)	
*NYHA classification / n (%)*	382			0.404
NYHA II		110 (28.8)	109 (28.5)	
NYHA III		79 (20.7)	74 (19.4)	
NYHA IV		8 (2.1)	2 (0.5)	
*Heart rate* ^a^	382	77 (±15.1)	75 (±13.8)	0.252
*LBBB*	382	20 (10.2)	22 (11.9)	0.587
*Heart rhythm at baseline*	382			
Sinus rhythm		96 (48.7)	113 (61.1)	0.015
Atrial fibrillation		62 (31.5)	35 (18.9)	0.007
Pacemaker rhythm		36 (18.3)	35 (18.9)	0.817
Other		3 (1.5)	2 (1.1)	n.a.
*Medication*				
ACE inhibitors	378	113 (57.9)	104 (56.8)	0.826
ATII antagonists	373	67 (34.7)	56 (31.1)	0.459
Beta-blockers	379	161 (82.6)	149 (81.0)	0.689
Digoxin	372	46 (23.8)	45 (25.1)	0.770
Diuretics	380	170 (86.3)	163 (88.1)	0.783
Nitrates	376	64 (33.2)	72 (39.3)	0.212
Statins	377	111 (57.5)	107 (58.2)	0.900
Coumarins	377	119 (61.0)	95 (52.2)	0.084
ASA	373	60 (31.1)	71 (39.4)	0.091

Values are presented as number (%) or mean ± SD

*ACE* angiotensin-converting enzyme, *ATII* angiotensin II, *ASA* acetylsalicylic acid, *LBBB* left bundle branch block, *NYHA* New York Heart Association functional classification

^a^Median (IQR 25-75)

### Primary endpoint

Functional status measured in METS remained unchanged in the intervention group, whereas it decreased in the control group over time. The changes over time were in favour of the intervention group (regression coefficient 0.318; *p*-value = 0.01). For VAS (*p* = 0.95), EQ-5D (*p* = 0.83) and Borg (*p* = 0.22), no differences over time between intervention and control group could be found (Tab. 2).

**Table 2:** Mean scores (imputed) of VAS, EQ-5D, METS and Borg at time of inclusion (T0) and on completing the study after 12 months (T12) with results of Poisson regression analysis per diagnostic test at T12

Group	Score T0 (SD)	Score T12 (SD)	∆ T12-T0	Coefficient intervention group	*p*-value
	VAS	VAS			
Control (*n* = 173)	57.82 (18.45)	63.18 (17.55)	5.36		
Intervention (*n* = 179)	63.31 (16.84)	66.03 (15.34)	2.72	−0.081	0.95
	EQ-5D	EQ-5D			
Control (*n* = 173)	0.61 (0.30)	0.63 (0.30)	0.02		
Intervention (*n* = 179)	0.64 (0.30)	0.65 (0.28)	0.01	0.005	0.83
	METS	METS			
Control (*n* = 173)	4.10 (1.79)	3.91 (1.70)	−0.19		
Intervention (*n* = 179)	4.35 (1.82)	4.36 (1.86)	0.01	0.318	0.01
	Borg	Borg			
Control (*n* = 173)	10.76 (3.76)	10.81 (3.74)	0.05		
Intervention (*n* = 179)	10.25 (3.71)	10.18 (3.62)	−0.07	−0.368	0.22

*Borg* Borg rating of perceived exertion scale, *EQ-5D* EQ-5D utility scores, *METS* metabolic equivalent scores, *n* numbers, *p* < 0.05 was considered statistically significant, *SD* standard deviation, *VAS* visual analogue scale

### Secondary endpoint

High VAS, EQ-5D utility and METS scores at baseline (T0) increased survival probability after 12 months (T12); the same was true for a low Borg score (Tab. 3). The hazard ratio for each point increase in METS for survival was 0.73 (95% CI 0.58–0.93), whereas the hazard ratio for Borg was 1.21 (95% CI 1.11–1.31). The hazard ratios for VAS and EQ-5D were 0.98 and 0.20, respectively (95% CI 0.96–0.99 and 0.07–0.54 respectively).

**Table 3:** Regression analysis of the relationship between functional status and quality of life at baseline and clinical outcome after 12 months of follow-up

Independent variable	Dependent variable	Coefficient estimate	*p*-value
VAS_T0	HF outpatient clinic visits (*n*)	−0.003	0.242
	HF hospital admission (days)	−0.002	0.344
	HF total hospital admissions (*n*)	−0.012	0.251
	Survival	−0.023	<0.05
EQ-5D_T0	HF outpatient clinic visits (*n*)	−0.282	<0.05
	HF hospital admission (days)	−0.297	<0.05
	HF total hospital admissions (*n*)	−0.384	0.528
	Survival	−1.623	<0.05
METS_T0	HF outpatient clinic visits (*n*)	−0.062	<0.05
	HF hospital admission (days)	−0.022	0.416
	HF total hospital admissions (*n*)	−0.188	0.173
	Survival	−0.311	<0.05
Borg_T0	HF outpatient clinic visits (*n*)	0.044	<0.05
	HF hospital admission (days)	0.051	<0.05
	HF total hospital admissions (*n*)	0.012	0.725
	Survival	0.186	<0.05

*Borg_T0* Borg rating of perceived exertion scale at baseline, *EQ-5D_T0* EQ-5D utility scores at baseline, *HF* heart failure, *METS_T0* metabolic equivalent scores at baseline, *n* numbers, *p* < 0.05 was considered statistically significant, *VAS_T0* visual analogue scale at baseline

## Discussion

In the TEHAF study, tailored telemonitoring stabilised the capacity of HF patients to perform physical activity in daily life but did not influence HRQoL. Better HRQoL and functional status at baseline were associated with better survival probability at T12.

### Functional status

Functional status is an important outcome in HF patients given that exercise capacity is one of the key determinants affecting daily-life activities in these patients. In addition, it is a strong predictor of mortality in patients with cardiovascular disease [21]. Therefore, improving physical activity is an important aim to achieve in treating patients with HF.

This study showed that METS remained stable over time in the study group, whereas a decline of METS was observed in the control group. At the same time, patients in the intervention group had 50% fewer routine face-to-face contacts with a HF specialist. It can be hypothesised that the education program of the Health Buddy stimulates participants to improve their exercise capacity. Another possible explanation could be the fact that telemonitoring increased adherence to pharmacological and non-pharmacological treatment in this study population [7]. Also, self-care increased in the telemonitoring group. Both increased therapy adherence and self-care may have positively influenced cardiopulmonary fitness. Nevertheless, the TEHAF study was not designed for this outcome, making these findings very likely due to chance.

Giordano et al. showed that the application of a home-based telesurveillance program improved functional status (measured by NYHA class and left ventricular ejection fraction) and physical performance (measured by 6‑min walk) in patients with HF [22]. Instead of focusing on patient education, their program was mainly used for remote monitoring by collecting vital parameters and regular telephone consultations with a HFN. Remote monitoring was also integrated in the Health Buddy system and may be the most important component of telemonitoring in improving functional status in HF patients.

Skobel et al. showed that a remote-controlled exercise program was more effective in increasing functional status than conventional training methods [23]. Therefore, more comprehensive telemonitoring programs may help to further improve the functional capacity of HF patients, but this needs to be properly studied in prospective trials.

### Quality of life

Improving the perceived burden of physical activity is important to HF patients as many feel tired, fatigued and unable to perform physical tasks. Furthermore, there is some discrepancy between objective measurement (e.g. cardiopulmonary exercise tests) and symptom perception (e.g. NYHA class) [24]. Therefore, it might be most important to improve the subjectively perceived burden of a particular domain to positively influence the patient’s well-being.

Using telemonitoring, no improvement of HRQoL in HF patients could be found in our study. This is consistent with the results of Balk et al., who used a TV-based telemonitoring program to improve HRQoL in HF patients [25]. Their intervention consisted of providing education and monitoring symptoms and behaviour, which made their results comparable to those of our study. A recently published randomised controlled trial (RCT) compared use of an e‑health adjusted care pathway and the heartfailurematters.org website to usual care in HF patients. No effect on HRQoL after 12 months of follow-up could be found [26].

### Additional endpoints

Functional status and HRQoL at baseline predicted survival after 1 year of follow-up. It has been shown previously that METS [27], EQ-5D and VAS [28] can predict survival in HF patients. Therefore, these scores may be valuable in assessing patients’ prognosis during post-clinical follow-up.

### Limitations

In our METS questionnaire, the highest obtainable score was 8.0. Therefore, exercise capacity of very fit patients may be underestimated. However, only one patient achieved a METS of 8.0. The original TEHAF study was underpowered to detect differences in functional status or HRQoL; thus, our findings are only hypothesis-generating. Furthermore, the control group was well treated, making the detection of clinical effects of the intervention difficult. We did not use HF-specific questionnaires and no questionnaires were used to provide detailed information on all relevant domains of HRQoL. Also, our analysis was done according to the per protocol principle, which could have led to a selective exclusion of patients. Therefore, attrition bias is possible. Still, the numbers of patients that did not finish the study were comparable between the two groups.

In this study, we used the EQ-5D to measure HRQoL. Other instruments such as the Minnesota Living With Heart Failure questionnaire were developed more specifically for HF patients. Still, the EQ-5D is an appropriate tool to measure HRQoL. Also, a more general questionnaire like the EQ-5D has a broader applicability because comparison among different medical conditions and patient groups is possible.

## Conclusion

In our study, tailored telemonitoring stabilised the functional status of HF patients. Therefore, telemonitoring may be a valuable tool to prevent deterioration of exercise capacity. However, because our study is a reanalysis of a RCT, this should be considered hypothesis-generating. Tailored telemonitoring did not improve HRQoL in HF patients. Further, sufficiently powered studies are needed to determine to what extent telemonitoring can improve functional status and HRQoL in HF patients.

## Caption Electronic Supplementary Material

Questionnaire consisting of 39 items to obtain METS
