# Supplementary material for: Effects of tailored telemonitoring on functional status and health-related quality of life in patients with heart failure
Source: Neth Heart J. 2019 Aug 14;27(11):565–74. doi: 10.1007/s12471-019-01323-x (PMC6823399; doi:10.1007/s12471-019-01323-x)
Supplement: Supplementary file 1 — Questionnaire consisting of 39 items to obtain METS [file 12471_2019_1323_MOESM1_ESM.docx]

Electronic Supplementary Material

METS per question

| Item | Question | METS |
| --- | --- | --- |
| 1 | Can you sleep a whole night without waking up because of dyspnea? | 0.9 |
| 2 | Can you listen to music or watch TV for 1 hour while lying down? | 1.0 |
| 3 | Can you listen to music or watch TV for 1 hour while sitting? | 1.0 |
| 4 | Can you talk or read for 30 minutes while sitting? | 1.3 |
| 5 | Can you go to the bathroom on your own? | 2.0 |
| 6 | Can you wash and dry yourself without ceasing? | 2.0 |
| 7 | Can you brush your hair without ceasing? | 2.5 |
| 8 | Can you walk for 5-10 minutes without stopping? | 2.5 |
| 9 | Can you cook without stopping? | 2.0 |
| 10 | Can you make your bed? | 2.0 |
| 11 | Can you do your groceries by yourself? | 2.3 |
| 12 | Can you water your plants non-stop? | 2.5 |
| 13 | Can you dust your house for 5-10 minutes non-stop? | 2.5 |
| 14 | Can you walk down the stairs (2 floors)? | 3.0 |
| 15 | Can you vacuum non-stop for 5-10 minutes? | 3.5 |
| 16 | Can you scrub non-stop for 5-10 minutes? | 3.5 |
| 17 | Can you swipe your driveway non-stop for 5-10 minutes? | 4.0 |
| 18 | Can you rake non-stop for 5-10 minutes? | 4.0 |
| 19 | Can you prune non-stop for 5-10 minutes with a hedge? | 4.5 |
| 20 | Can you prune non-stop for 5-10 minutes with an electrical hedge? | 3.5 |
| 21 | Can you do pull the weeds out non-stop for 5-10 minutes? | 4.5 |
| 22 | Can you seed non-stop for 5-10 minutes? | 4.5 |
| 23 | Can you paint your house non-stop for 5-10 minutes? | 4.5 |
| 24 | Can you move wooden blocks non-stop for 5-10 minutes? | 5.0 |
| 25 | Can you walk up the stairs (2 floors)? | 5.0 |
| 26 | Can you cut the grass non-stop for 5-10 minutes? | 5.5 |
| 27 | Can you ride a bike non-stop for 10-15 minutes? | 6.0 |
| 28 | Can you ride a bike with a power of 100 Watt? | 6.0 |
| 29 | Can you ride a bike with a power of 50 Watt? | 4.0 |
| 30 | Can you golf with a golf cart non-stop for 30 minutes? | 3.5 |
| 31 | Can you golf without a golf cart non-stop for 30 minutes? | 4.5 |
| 32 | Can you swim laps non-stop for 10 minutes? | 7.0 |
| 33 | Can you swim non-stop for 10 minutes (no laps)? | 4.0 |
| 34 | Can you play tennis non-stop for 15 minutes (doubles match only)? | 5.0 |
| 35 | Can you play tennis non-stop for 15 minutes (singles match)? | 8.0 |
| 36 | Can you go jogging non-stop for 10 minutes? | 6.0 |
| 37 | Can you run faster than 8 km/h non-stop for 10 minutes? | 8.0 |
| 38 | Can you ski downhill non-stop? | 7.0 |
| 39 | Can you walk an up-and-down a road non-stop for 15 minutes? | 7.0 |

METS = Metabolic equivalent scores
